# HIF-1 mediated activation of antimicrobial peptide LL-37 in type 2 diabetic patients

**DOI:** 10.1007/s00109-021-02134-7

**Published:** 2021-10-15

**Authors:** Soumitra Mohanty, Witchuda Kamolvit, Silvia Zambrana, Eduardo Gonzales, Jonas Tovi, Kerstin Brismar, Claes-Göran Östenson, Annelie Brauner

**Affiliations:** 1 Department of Microbiology, Tumor and Cell Biology, Division of Clinical Microbiology, Karolinska Institutet and Karolinska University Hospital, 17176 Stockholm, Sweden; 2 Department of Molecular Medicine and Surgery, Karolinska Institutet, Stockholm, Sweden; 3 Area de Farmacologia, Facultad de Ciencias Farmacéuticas Y Bioquimicas, Instituto de Investigaciones Farmaco Bioquimicas, Universidad Mayor de San Andres, La Paz, Bolivia; 4 Capio Health Care Center, Solna, Sweden; 5 Faculty of Medicine Siriraj Hospital, Mahidol University, Bangkok, Thailand

**Keywords:** HIF-1, LL-37, Cytokines, Type 2 diabetes, Urinary tract infections

## Abstract

**Abstract:**

Infections are common in patients with diabetes, but increasing antibiotic resistance hampers successful bacterial clearance and calls for alternative treatment strategies. Hypoxia-inducible factor 1 (HIF-1) is known to influence the innate immune defense and could therefore serve as a possible target. However, the impact of high glucose on HIF-1 has received little attention and merits closer investigation. Here, we show that higher levels of proinflammatory cytokines and *CAMP*, encoding for the antimicrobial peptide cathelicidin, LL-37, correlate with HIF-1 in type 2 diabetic patients. Chemical activation of HIF-1 further enhanced LL-37, IL-1β, and IL-8 in human uroepithelial cells exposed to high glucose. Moreover, HIF-1 activation of transurethrally infected diabetic mice resulted in lower bacterial load. Drugs activating HIF-1 could therefore in the future potentially have a therapeutic role in clearing bacteria in diabetic patients with infections where antibiotic treatment failed.

**Key messages:**

• Mohanty et al. “HIF-1 mediated activation of antimicrobial peptide LL-37 in type 2 diabetic patients.”

• Our study highlights induction of the antimicrobial peptide, LL-37, and strengthening of the innate immunity through hypoxia-inducible factor 1 (HIF-1) in diabetes.

• Our key observations are:

1. HIF-1 activation increased LL-37 expression in human urothelial cells treated with high glucose. In line with that, we demonstrated that patients with type 2 diabetes living at high altitude had increased levels of the LL-37.

2. HIF-1 activation increased IL-1β and IL-8 in human uroepithelial cells treated with high glucose concentration.

3. Pharmacological activation of HIF-1 decreased bacterial load in the urinary bladder of mice with hereditary diabetes.

• We conclude that enhancing HIF-1 may along with antibiotics in the future contribute to the treatment in selected patient groups where traditional therapy is not possible.

**Supplementary Information:**

The online version contains supplementary material available at 10.1007/s00109-021-02134-7.

## Introduction

Patients with diabetes mellitus have an increased risk of infections, especially urinary tract infections, often recurrent and with enhanced severity [1]. Moreover, the heightened antibiotic resistance is a global threat, and novel treatment strategies are therefore mandatory.

The innate immune response including antimicrobial peptides and cytokines produced by uroepithelial cells plays an important role in preventing infections. Hypoxia-inducible factor 1 (HIF-1) is a major regulator of cellular adaptation to low oxygen conditions. Its activity is dependent on the degradation of the HIF-1α subunit in normoxia regulated by O_2_ dependent hydroxylation of the proline residue by prolyl hydroxylase domain protein 2 (PHD2). This promotes binding of the von Hippel-Lindau protein (VHL), leading to ubiquitination and proteasomal degradation, whereas inactivation of PHD2 activates the HIF-1 signaling [2, 3]. HIF-1 has been studied in normoglycemic conditions and is involved in regulating inflammation and production of antimicrobial peptides [4], in particular LL-37 [5, 6], an antibacterial peptide, inactivating drug-sensitive and resistant bacteria by forming discrete membrane lesions and inhibiting the cell wall, nucleic acid, and protein biosynthesis [7–9]. A major difference to conventional antibiotics is that resistance seldom develops, making antimicrobial peptides a possible novel complement to treat specific infections [10]. Interestingly, in acute pyelonephritis, metabolic acidosis stimulates HIF-1 which further induces LL-37 expression in the renal collecting ducts [11]. Mice lacking HIF-1α in their myeloid lineage showed less bactericidal activity when infected with group A streptococci and had less ability to prevent systemic bacterial spread [12]. In addition, HIF-1 was shown to have effect on uropathogenic *Escherichia coli* infection [5]. Although hypoxia alone can induce HIF-1 activation, high glucose levels, as can be found in diabetes, is known to impair the stability and function of HIF-1 [3, 13], but the impact of glucose on the immune response and infections is less well studied, in spite of the increased risk of serious complications during hyperglycemia. It is worthy of note that urinary tract infections may even be life-threatening in patients with diabetes. Exploring the effect during hyperglycemia of HIF-1 on infections could therefore open new avenues of treatment. Pharmacological inducers activating HIF-1, like dimethyloxalylglycine (DMOG) or deferoxamine (DFO), have been shown to partly restore the HIF-1 function in diabetes [14] and increase the expression of phosphoglycerate kinase 1 (PGK1). This promotes glycolysis using the AKT signaling pathway [15] which also plays an important role in insulin signaling and expression of antimicrobial peptides [16] and may therefore possibly also indirectly affect bacterial clearance.

To fill the knowledge gap, we evaluate the possible impact of high altitude on innate immune response, and to assess the option to enhance the epithelial defense against invading uropathogenic *E. coli* in diabetes, we investigated the potential effect of activating HIF-1 under hypoxic and high glucose conditions in vitro as well as in diabetic mice and in patients with type 2 diabetes.

## Methods

### Collection of urine and serum from patients with type 2 diabetes

Urine and venous blood samples from patients with type 2 diabetes were collected separately at outpatient clinics and taken care of at Karolinska University Hospital Laboratory and Universidad Mayor de San Andres between 2019 and 2020 and stored at − 80 °C prior to analysis. Urinary cells were immediately harvested for total RNA extraction. Nine out of twenty-three patients in La Paz were male, median age 61 (44–74) years, BMI 30.5 (22.5–40.8) kg m^−2^, and HbA1c 56 (36–116) mmol mol^−1^. Twenty-one out of twenty-five in Stockholm were male with median age 67 (44–80) years, BMI 30.3 (24–40.2) kg m^−2^, HbA1c 50 (40–73) mmol mol^−1^. Patients with known co-morbidities, like recurrent urinary tract infections, or on estrogen therapy were excluded. A detailed description of clinical parameters is given in Table 1.

**Table 1:** Clinical parameters of patients included in the study. Higher HbA1c and urine glucose levels were observed in patients living at high compared to low altitude. *ACE* angiotensin-converting enzyme; *ARB* angiotensin receptor blocker; *DPP4 inhibitor* dipeptidylpeptidase-4 inhibitor; *GLP-1 agonists* glucagon-like peptide-1 agonists; *NOAC* non-vitamin K antagonist oral anticoagulants; *SGLT2 inhibitor* sodium-glucose cotransporter-2 inhibitor. Statistics was performed using unpaired test comparing persons living at low vs high altitude. Significance levels mentioned as *P < 0.05

	**Sl. no**	**Type**	**Age**	**Gender**	**BMI** **(kg m**^**−2**^**)**	**HbA1c** **(mmol mol**^**−1**^**)**	**Urine glucose** **(mmol l**^**−1**^**)**	**Antidiabetic treatment**	**Blood pressure treatment**	**Dyslipidemia treatment**	**Other treatment**
**Low altitude**	1	T2D	44	M	32.6	61	< 0.7	DPP4 inhibitor, insulin		Statins	
	2	T2D	75	F	29.1	45	0.7	Diet	ARB, beta blocker		Thyroxin
	3	T2D	62	M	38.7	66	7	Metformin, GLP-1 agonists, insulin		Statins	
	4	T2D	64	M	30.3	47	< 0.7	Metformin	ACE inhibitor, beta blocker	Statins	
	5	T2D	67	M	27.7	50	1	Metformin, DPP4 inhibitor	ACE inhibitor, calcium blocker	Statins	NOAC
	6	T2D	58	F	32	50	< 0.7	GLP-1 agonists, insulin			
	7	T2D	64	M	25	49	< 0.7	DPP4 inhibitor		Statins	Thrombocyte aggregation inhibitor
	8	T2D	69	M	34.2	51	< 0.7	Metformin, DPP4 inhibitor	ARB, calcium blocker	Statins	Antacid
	9	T2D	65	M	26.8	53	0.9	Metformin		Statins	Dopamine agonist
	10	T2D	75	M	33.3	46	< 0.7	Metformin, GLP-1 agonists		Statins	
	11	T2D	72	M	30.5	73	0.9	Insulin	ARB, calcium blocker	Statins	Thrombocyte aggregation inhibitor
	12	T2D	51	M	28.1	46	< 0.7	Metformin			
	13	T2D	68	M	33.2	53	< 0.7	Metformin			
	14	T2D	80	M	34.7	59	< 0.7	Glitazon	ARB, ACE inhibitor, spironolactone, thiazide, calcium blocker	Statins	Thrombocyte aggregation inhibitor
	15	T2D	76	M	29.9	55	< 0.7	Metformin, DPP4 inhibitor, sulfonylurea	ARB, calcium blocker		Thrombocyte aggregation inhibitor
	16	T2D	74	M	32.7	51	< 0.7	DPP4 inhibitor	Beta blocker		
	17	T2D	61	M	32.2	45	1	Metformin, insulin			
	18	T2D	52	M	26.2	50	< 0.7	Insulin		Statins	
	19	T2D	52	M	30.3	40	0.7	Diet	ACE inhibitor, calcium blocker		Thrombocyte aggregation inhibitor
	20	T2D	65	M	40.2	59	1	Metformin			
	21	T2D	68	M	29.1	41	< 0.7	Metformin			
	22	T2D	79	M	24	47	< 0.7	Metformin		Statins	
	23	T2D	63	F	22.5	42	0.7	Diet		Statins	
	24	T2D	77	M	26.2	61	2	Metformin, Insulin			
	25	T2D	72	F	27.5	47	< 0.7	Metformin, GLP-1 agonists		Statins	
**High altitude**	1	T2D	59	F	36.5	101	60	Metformin			
	2	T2D	66	F	23.6	51	< 0.7	Metformin	ACE inhibitor, beta blocker	Gemfibrozil	
	3	T2D	51	F	35.2	40	< 0.7	Metformin, sulfonylurea			
	4	T2D	72	F	22.5	116	294	Metformin			
	5	T2D	54	F	40.8	36	10	Metformin	Beta receptor blocker		Thyroxine
	6	T2D	52	M	23.1	39	2	Metformin			
	7	T2D	53	F	26.6	64	< 0.7	Metformin			
	8	T2D	65	F	25.1	96	133	Metformin, sulfonylurea, insulin			
	9	T2D	63	F	32.1	85	< 0.7	Metformin, sulfonylurea			
	10	T2D	61	M	28.0	37	< 0.7	Sulfonylurea			
	11	T2D	49	F	25.1	115	28	Insulin	ACE inhibitor		
	12	T2D	74	M	27.4	49	2	Metformin	ACE inhibitor		
	13	T2D	65	F	27.4	39	< 0.7	Diet			
	14	T2D	53	F	31.7	64	< 0.7	Metformin, sulfonylurea	ACE inhibitor		
	15	T2D	67	F	31.8	88	461	Metformin, sulfonylurea	ACE inhibitor		
	16	T2D	44	M	37.4	38	< 0.7	Metformin		Statins	Thrombocyte aggregation inhibitor
	17	T2D	58	M	32.0	48	< 0.7	Metformin	ACE inhibitor		
	18	T2D	54	M	29.8	52	< 0.7	Metformin	Beta receptor blocker		
	19	T2D	64	F	34.5		< 0.7	Metformin	ACE inhibitor	Statins	
	20	T2D	55	M	32.2	41	0.8	Insulin	ACE inhibitor	Statins	
	21	T2D	61	M	30.5	60	284	Metformin, insulin		Statins	
	22	T2D	66	F	37.0	67	1	Metformin, insulin	ACE inhibitor		
	23	T2D	65	M	30.0	83	258	Metformin, insulin			
Stats						* (unpaired *t* test)	* (unpaired *t* test)				

### Bacterial strain and cell culture conditions

Uropathogenic *E. coli* strain CFT073 expressing type 1 and P and S fimbriae along with α-hemolysin was used in all experiments. For in vitro experiments, human uroepithelial cell lines, TERT-NHUC (kindly provided by M. A. Knowles, Leeds, UK), and 5637 (HTB-9, American Type Culture Collection) were used. To mimic hyperglycemia and high altitude, cells were exposed to 30 mM glucose and hypoxia (1% O_2_) as compared to normal glucose levels of either 5.5 or 6 mM and normoxia with 21% O_2._ Mannitol (Sigma) was used to verify the osmotic effect of glucose. For HIF-1 activation, 400 µM of HIF-hydroxylase inhibitor, 2-oxoglutarate analogue DMOG (Frontier Scientific) or 100 µM of iron chelator, DFO (Sigma) was used. DMOG effectively inhibits multiple 2OG dioxygenase and activates HIF-1 and might influence nucleic acid repair and fatty acid metabolism where 2OG dioxygenase is greatly involved [17]. During infection, DMOG was used to avoid possible influence on bacterial growth by the iron chelator, DFO.

### Mouse model of urinary tract infection

Mouse experiments were approved by the Northern Stockholm Animal Ethics Committee and experiments were carried out according to the guidelines of the Federation of Laboratory Animal Science Association and in compliance with the Committee’s requirements (10,370–2018). Ten-week-old female mice with hereditary diabetes, db/db (BKS (D)-*Leprdb*/JOrlRj) (Janvier Laboratories) with a median blood glucose of 24.9 (16.1–27.6) mM were used. The mice received DMOG (320 mg/kg) or DMSO intraperitoneally every second day for 7 days prior to infection. Mice were anaesthetized, transurethrally infected with 0.5 × 10^8^ colony-forming units (CFU) of *E. coli*, sacrificed after 24 h and 7 days infection. Urinary bladders were cut open, washed with PBS to remove non-adherent bacteria, and lysed in PBS using Dounce’s homogenizer. Total bacterial load was counted using CFU assay [18].

### Cell infection assay

TERT-NHUC and 5637 cells were pretreated with glucose (30 mM) with and without DMOG (400 µM) and incubated in hypoxia (1% O_2_) for at least 24 h followed by infection with 10^6^ *E. coli*. Cells were infected for 2 h with *E. coli* at MOI 10, washed with PBS to remove non-adherent bacteria, and supplemented with fresh medium for another 2 h. To avoid possible interference of bacterial killing by the released antimicrobial peptides, no antibiotics were used. Furthermore, cells were washed with PBS, lysed with 200 µl of 0.1% Triton X-100 in PBS (pH-7.4), and plated on blood agar plates. Bacterial load was calculated by a number of adhered and intracellular bacteria in relation to the total number of bacteria added from the same experiment.

### Reporter gene assay

To assay the effect of glucose on the transcriptional activity of HIF-1, we used a plasmid (pT81/HRE-luc) that contains three tandem copies of the erythropoietin hypoxia response element (HRE) in front of the herpes simplex thymidine kinase promoter and the luciferase gene [19]. Human uroepithelial cells 5637 cultured in 24-well plates were transfected at 70–80% confluence with the reporter plasmid (0.5 μg/well), after changing the medium to Optimem (Life Science Technologies), using the FuGENE 6 transfection reagent (Roche Diagnostics) according to the manufacturer’s instructions. After 6 h, the medium was changed to RPMI containing either 5.5 or 30 mM glucose. Cells were cultured in triplicate under hypoxic (1% O_2_) or normoxic conditions (21% O_2_). After 39 h of incubation, luciferase activity was determined as described by the manufacturer (Promega Biotec), and the reporter gene activity was standardized to protein content and expressed as fold induction relative to the activity under normoxic, normoglycemic conditions.

### qRT-PCR

After the required incubation, TERT-NHUC cells were directly processed for RNA isolation, whereas bladder tissues were homogenized manually with Dounce’s homogenizer. Total RNA was extracted using RNeasy mini kit (Qiagen) and transcribed to cDNA using a high-capacity cDNA synthesis kit (Applied Biosystems) according to the manufacturer’s protocol. Up to 0.5 µg of RNA was reverse transcribed using random primers for 10 min at 25 °C, 120 min at 37 °C, and inactivation at 85 °C for 5 min. Real-time PCR was performed after initial denaturation at 95 °C for 10 min, each cycle consisted of 15 s at 95 °C, 60 s at 60 °C (touchdown of 1 °C per cycle from 66° to 60 °C), and 30 s at 72 °C using standard SYBR green and Taqman (Applied Biosystems) protocol in a Rotor-Gene PCR cycler (Corbett Life Science) [18]. Primer and probe details are mentioned in Table 2. Melting curves were produced at the end of the run with SYBR green to ensure the specificity of the amplification. Relative expressions of target genes were presented as 2^−∆CT^ and fold change as 2^−∆∆CT^ compared to respective control.

**Table 2:** Primers/probes used in the study

**Primer**	**Forward (5′-3′)**	**Reverse (5′-3′)**	**Reference**
Human *CAMP*	ACCCAGCAGGGCAAATCT	GAAGGACGGGCTGGTGAA	[5]
Human *HIF1A*	GCTGGCCCCAGCCGCTGGAG	GAGTGCAGGGTCAGCACTAC	[45]
Human *PGK1*	AGTCCTTATGAGCCACCT	CAGAACATCCTTGCC CAG	[46]
Mouse *Pgk1*	AGTCCGTTGTCCTTATGAG	CAGAACATCCTTGCCCAG	This study
Human *IL1B*	CACGATGCACCTGTACGATCA	GTTGCTCCATATCCTGTCCCT	[47]
Human *CXCL8*	AAGAGAGCTCTGTCTGGACC	GATATTCTCTTGGCCCTTGG	[48]
Human *PBGD*	AGGATGGGCAACTGTACC	GTTTTGGCTCCTTTGCTCAG	[49]
Mouse *PBGD*	GTGTTGCACGATCCTGAAACT	GTTGCCCATCCTTTATCACTGTA	[49]
**Probe**			
Mouse *Cramp*	Mm00438285_m1		Invitrogen
Mouse *PBGD*	Mm01143545_m1		Invitrogen

### Immunofluorescence staining

Bladder sections were deparaffinized and rehydrated, pretreated with 0.3% Triton X-100 in PBS, or boiled in citrate buffer, 1 mM EDTA, 10 mM tris, 0.05% Tween 20 (pH 9). Cells were fixed in 4% PFA for either 5 or 30 min at room temperature and permeabilized with 0.1% Triton X-100 in PBS. Thereafter, cells were blocked with 5% BSA in PBS for 30 min; further sections were treated with FX Signal Enhancer (Invitrogen) at room temperature for 30 min to eliminate nonspecific fluorescence commonly seen with the application of fluorescent secondary antibody. To improve the specific fluorescence, sections were additionally blocked with the sera from the species in which the secondary antibodies were raised for 60 min. Cells were stained with primary anti-LL-37 [20] (1:200, Santacruz) and anti-HIF-1α [21] (1:100, BD) antibody in 1:1 ratio of 1 × PBS with 0.1% Tween 20 (PBS-T) and 5% BSA in PBS, sections with anti–*E. coli* (1:200; AbD Serotec), anti-UPIIIa (1:200; Santa Cruz) and incubated overnight at 4 °C. Both sections and cells were washed with 1 × PBS-T and further incubated with respective secondary Alexa Fluor–conjugated antibody (Invitrogen) in 1:600 (sections), 1:400, or 1:100 (cells) for 1 h at room temperature, followed by staining with DAPI for 15 min, washed and mounted in Fluoromount G (Southern Biotech). Slides were analyzed with a Zeiss 700 confocal microscope using 63 × oil immersion objective, from 3 to 5 random view fields per coverslip. Fluorescence intensity was quantified with the ImageJ software.

### ELISA

Human serum and cell-free culture from in vitro experiments were collected using standard protocol [18]. LL-37 (Hycult Biotech), IL-1β, and IL-8 (R&D Biosystems) were analyzed according to the manufacturer’s recommendations.

### Statistical analysis

All statistical tests were performed in Graph pad Prism version 5. Data were obtained from Students’ unpaired *t*-test, paired nonparametric two-tailed Wilcoxon matched-pairs, signed-rank test, and Pearson’s correlation as appropriate. Differences with *p* values below 0.05 were considered statistically significant.

## Results

### Activation of HIF-1 increases LL-37 expression in diabetes

Antimicrobial peptides are the first line to protect against invading microorganisms. We have previously demonstrated the importance of LL-37 in the human urinary tract [22]. However, the possible impact of HIF-1 on the antimicrobial peptide LL-37, encoded by *CAMP* in diabetes, is not fully understood and was therefore investigated.

Cells cultured in hypoxia at a normal glucose concentration (6 mM) showed increased expression of *CAMP* while this was not observed at high glucose (30 mM) or in normoxia (Fig. S1a). Furthermore, to investigate if high glucose levels affected the cells by its induced osmolarity, we exposed the cells to an identical high concentration of mannitol that is not metabolized by the exposed cells. We did not observe any difference with respect to *CAMP* induction between high glucose and high mannitol stimulated cells, suggesting that the observed effect, at least partly, was mediated by hyperosmolarity (Fig. S1b). It cannot, however, be ruled out, that glucose as present in persons with diabetes can exert an additive and substrate-specific effect on LL-37 levels.

**Fig. 1:**
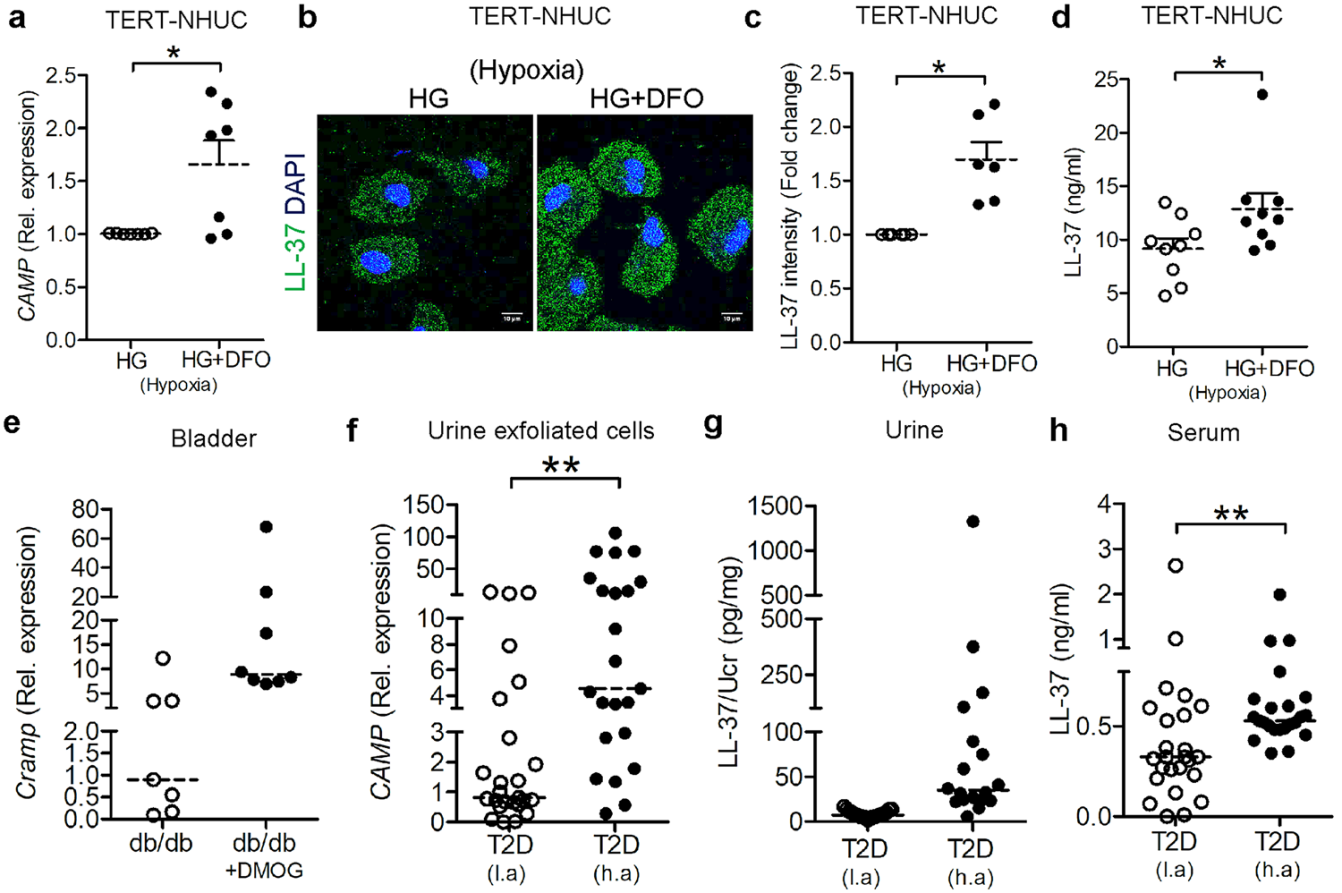
HIF-1 activation increased expression of the antimicrobial peptide, LL-37, in diabetes. TERT-NHUC cells, demonstrating expression of **a** *CAMP* mRNA (*n* = 7, unpaired *t* test) 24 h post-treatment. **b** Representative intracellular LL-37 stained (*n* = 6) with Alexa-488 (green) and DAPI (blue) for nucleus 36 h post-treatment. **c** Average fluorescence intensity LL-37 (*n* = 6, Wilcoxon matched-pairs signed-rank test). **d** Secreted LL-37 levels (*n* = 3, in triplicate, unpaired *t* test) after 36 h treatment with DFO in hypoxia. **e** *Cramp* mRNA in DMOG (*n* = 8), DMSO treated db/db mice (*n* = 7) urinary bladder 7 days post-*E. coli* infection (unpaired *t* test). **f** Human urine exfoliated cells (*n* = 25 and 23, respectively, unpaired *t* test) were analyzed for *CAMP* mRNA. **g** and human urine (*n* = 19 and 18, respectively, unpaired *t* test) for LL-37, normalized to urine creatinine (Ucr) and **h** serum (*n* = 25, unpaired *t* test) from patients with type 2 diabetes (T2D) living at high altitude, La Paz, Bolivia, 3600 m above sea level, (h.a) and low altitude, Stockholm, Sweden, 28 m above sea level, (l.a) were analyzed for LL-37 protein. In vitro analysis was performed in either duplicate or triplicate. Average values are shown for each set. High glucose (HG). Data are shown as mean ± SEM. Results from patients and mice are presented as median. Significance levels mentioned as *P < 0.05, **P < 0.01, and ****P < 0.0001

To activate the HIF-1, uroepithelial cells cultured in hypoxia and exposed to high glucose levels were treated with DFO. Indeed, treatment with DFO was followed by significantly increased *CAMP* mRNA (Fig. 1a), total intracellular LL-37 protein located in the cytoplasm, and within vesicles (Fig. 1b, c) as well as secreted LL-37 (Fig. 1d). To avoid the possible influence of DFO on *E. coli*, DMOG was used, which in addition has not been shown to affect blood glucose levels [23]. Likewise, diabetic, db/db female mice infected with *E. coli* and pretreated with HIF-1 activator DMOG also showed a trend of increased expression of *Cramp* (Fig. 1e), while no difference was observed between uninfected mice with and without DMOG treatment (Fig. S1c, d). To mimic the activation of HIF-1, type 2 diabetic patients living at high altitude, La Paz, Bolivia (3600 m above sea level, masl), were included together with a corresponding patient group living at low altitude, Stockholm, Sweden (28 masl), serving as controls. Interestingly, in samples from patients living at high altitude, *CAMP* expression was higher in urine exfoliated cells (Fig. 1f) After normalization to urine creatinine, LL-37 was detected in urine samples in seven out of 18 patients, but not from any of the patients living at low altitude (Fig. 1g). Moreover, higher serum LL-37 levels were detected in samples from patients living at high altitude (Fig. 1h) with no difference in the expression between male and female irrespective of altitude. To rule out a possible effect of different antidiabetic therapies on *HIF1* and *CAMP*, results were evaluated based on the treatment given, irrespective of altitude. No difference was observed between patients treated with insulin (*n* = 12) or drugs affecting the insulin signaling pathway, i. e., sulfonylurea and GLP-1 agonists (*n* = 8) or those treated with other oral antidiabetic drugs such as metformin, glitazon, DPP4, and SGLT2 inhibitors (*n* = 27) (Fig. S2a–b, Table 1).

### Chemical activation of HIF-1 and high altitude increases HIF-1 target genes in diabetes

**Fig. 2:**
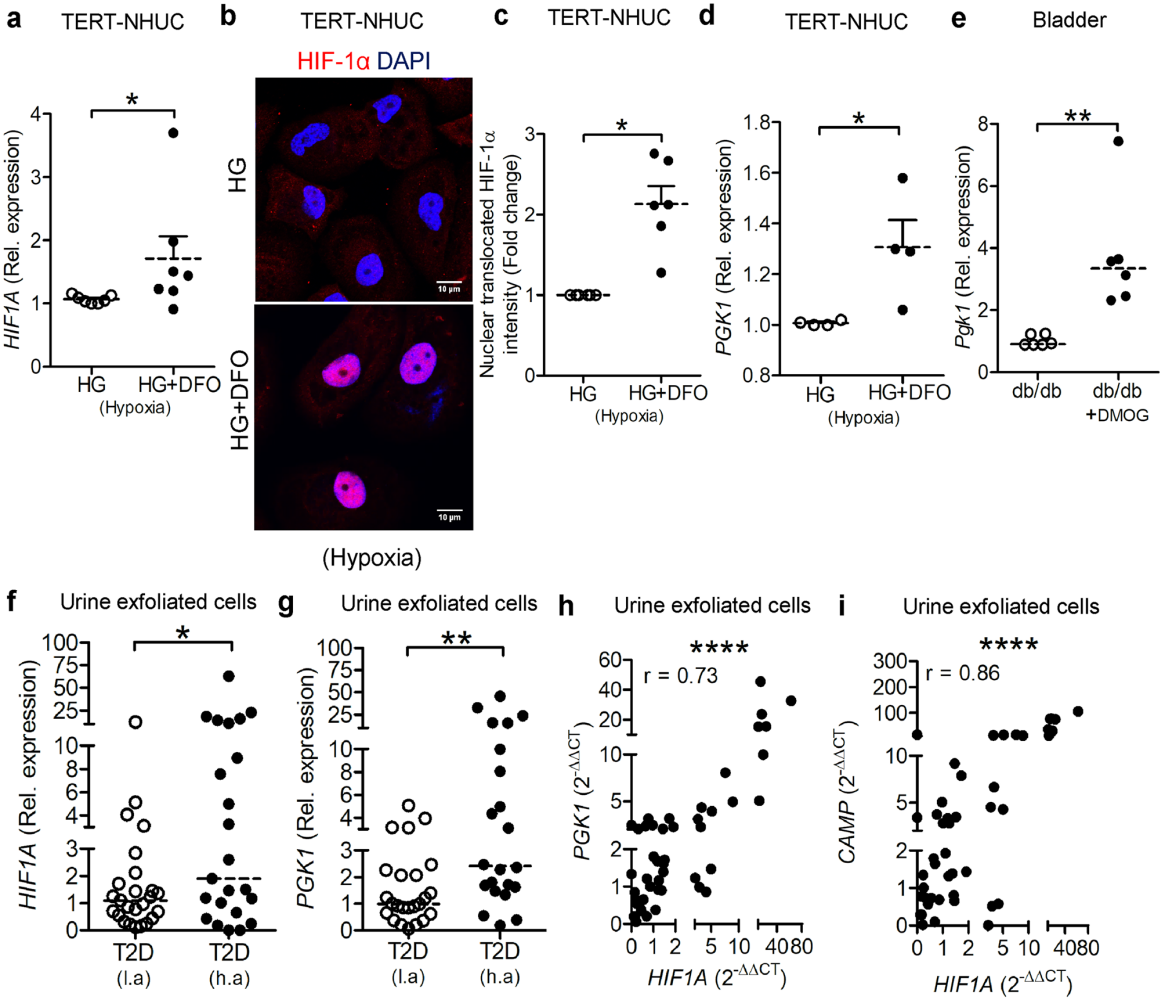
Hypoxia and chemical activation of HIF-1 activates HIF target genes in diabetic urinary bladder. In TERT-NHUC cells, **a** expression of *HIF1A* mRNA (*n* = 7, unpaired *t* test) 24 h post-treatment. **b** Nuclear translocation of HIF-1α stained (*n* = 6) with Alexa-594 (red) and DAPI (blue) 36 h post-treatment, **c** nuclear HIF-1α intensity (*n* = 7, Wilcoxon matched-pairs signed-rank test), and **d** *PGK1* mRNA (*n* = 4, unpaired *t* test) 24 h post-treatment. **e** *Pgk1* expression in 7 days DMOG pretreated uninfected diabetes db/db mice urinary bladder (*n* = 6, unpaired *t* test). **f** *HIF1A* **g** *PGK1* mRNA from urine exfoliated cells from patients with type 2 diabetes (T2D) (*n* = 25 and 23, respectively, unpaired *t* test) living at high altitude, La Paz, Bolivia, 3600 m above sea level, (h.a) and low altitude, Stockholm, Sweden, 28 m above sea level, (l.a). Correlation graph showing expression of **h** *PGK1* and **i** *CAMP* versus *HIF1A* (*n* = 25 and 23, respectively, Pearson’s correlation coefficient)*. *In vitro experiments were performed in either duplicate or triplicate. Average values are shown for each set. High glucose (HG). Data are shown as mean ± SEM. Results from patients and mice are presented as median. Significance levels mentioned as *P < 0.05, **P < 0.01, and ****P < 0.0001

HIF-1 signaling triggers multiple genes, many of which are considered as HIF-1 target genes participating in the positive regulation of the HIF-1 function [24]. HIF-1also regulates the expression of several genes in diabetes [25]. To confirm the effect of high glucose on HIF-1, we transiently transfected uroepithelial cells 5637 with a HRE-driven luciferase reporter gene, which generates a hypoxia-dependent activation response [3]. Exposure of the cells to high glucose concentrations, under hypoxic conditions, produced slightly, although not significantly, lower activation (Fig. S3). To activate *HIF1A*, TERT-NHUC cells exposed to high glucose, hypoxia together with DFO, showed significantly increased *HIF1A* mRNA (Fig. 2a). The stability of HIF-1α was confirmed by its nuclear translocation (Fig. 2b, c) and increased expression of *PGK1* (Fig. 2d). Similarly, DMOG-treated diabetic mice also showed increased *Pgk1* mRNA in relation to non-treated mice (Fig. 2e). Interestingly, we observed significantly increased expression of *HIF1A* (Fig. 2f) and HIF-1 target gene *PGK1* (Fig. 2g) in exfoliated urine cells from type 2 diabetic patients living at high altitude. Moreover, we observed a direct correlation of *PGK1* (Fig. 2h) and *CAMP* (Fig. 2i) with *HIF1A* mRNA.

### HIF-1 activation increases proinflammatory cytokines in diabetes

**Fig. 3:**
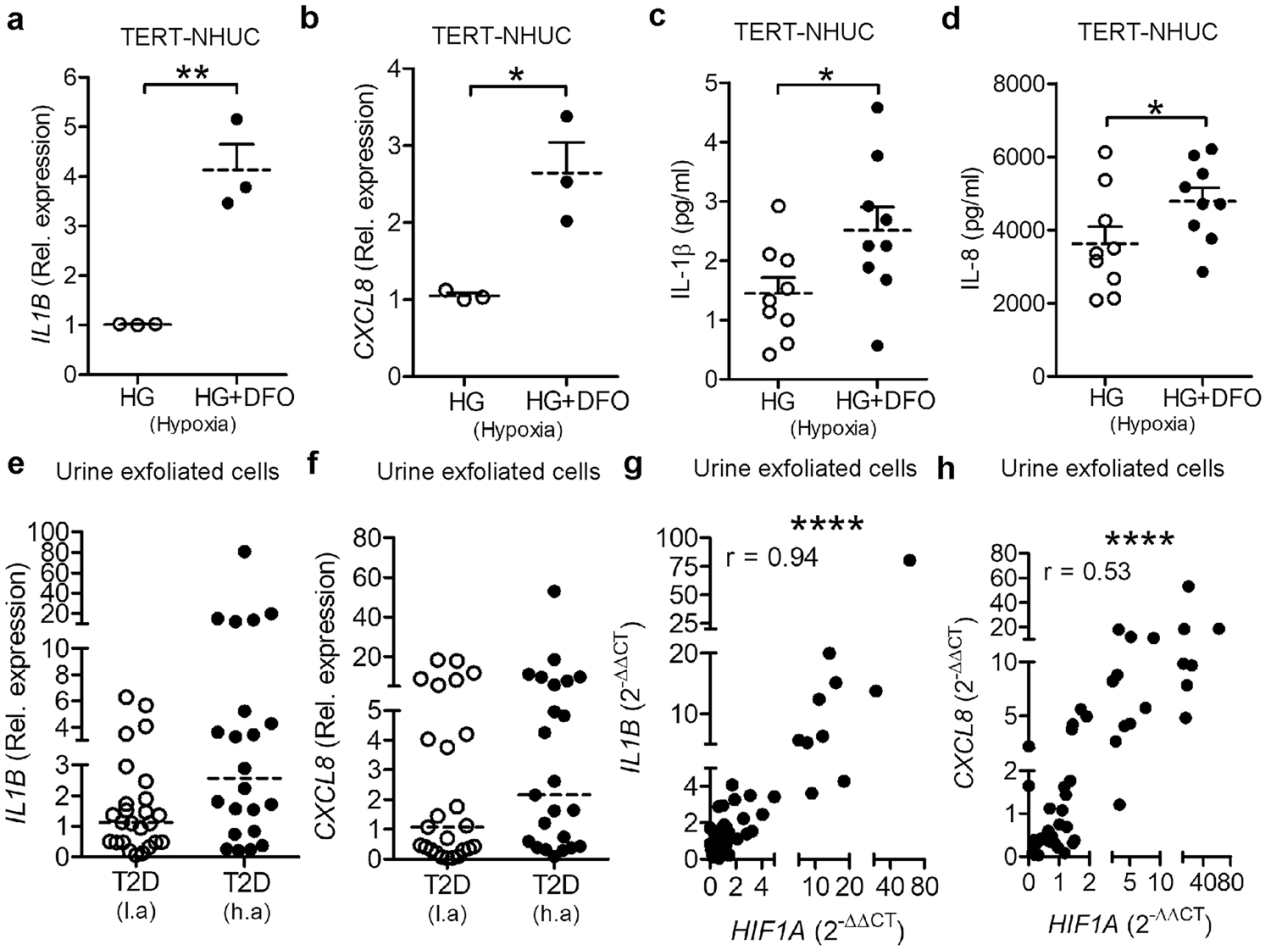
HIF-1 activation increased proinflammatory cytokines. TERT-NHUC cells expression of **a** *IL1B* **b** *CXCL8* mRNA (*n* = 3, unpaired *t* test) 24 h post-treatment, **c** IL-1β and **d** IL-8 protein (*n* = 3, in triplicate, unpaired *t* test) 36 h post-treatment with DFO in hypoxia. Urine exfoliated cells from patients with type 2 diabetes (T2D) patients living at high altitude in La Paz, Bolivia, 3600 m above sea level (h.a) and low altitude, Stockholm, Sweden, 28 m above sea level (l.a); (*n* = 25 and 23, respectively, unpaired *t* test), were analyzed for **e** *IL1B* and **f** *CXCL8*. Correlation graph showing expression of **g** *IL1B* versus *HIF1A* and **h** *CXCL8* versus *HIF1A* (*n* = 25 and 23, respectively, Pearson’s correlation coefficient)*. *In vitro experiments were performed in triplicate. Average values are shown for each set. High glucose (HG). Data are shown as mean ± SEM. Results from patients are presented as median. Significance levels mentioned as *P < 0.05, ***P < 0.001, and ****P < 0.0001

HIF-1 is known to be a positive regulator of cytokines namely IL-1A, IL-1B, and IL-6 in non-diabetic patients [26]. Cytokines are also involved in neutrophil infiltration, which helps in bacterial clearance [27]. Treatment with DFO of TERT-NHUC cells exposed to high glucose significantly upregulated *IL1B* (Fig. 3a) and *CXCL8* (Fig. 3b) mRNA as well as secreted IL-1β (Fig. 3c) and IL-8 (Fig. 3d) protein levels. Similarly, the expression of the proinflammatory cytokine *IL1B* mRNA (Fig. 3e) and *CXCL8* (Fig. 3f) showed a clear trend of increase with a high correlation to *HIF1A* mRNA levels in samples from type 2 diabetic patients living at high altitude (Fig. 3g, h).

### HIF-1 activation protects the host from E. coli infection in diabetes

**Fig. 4:**
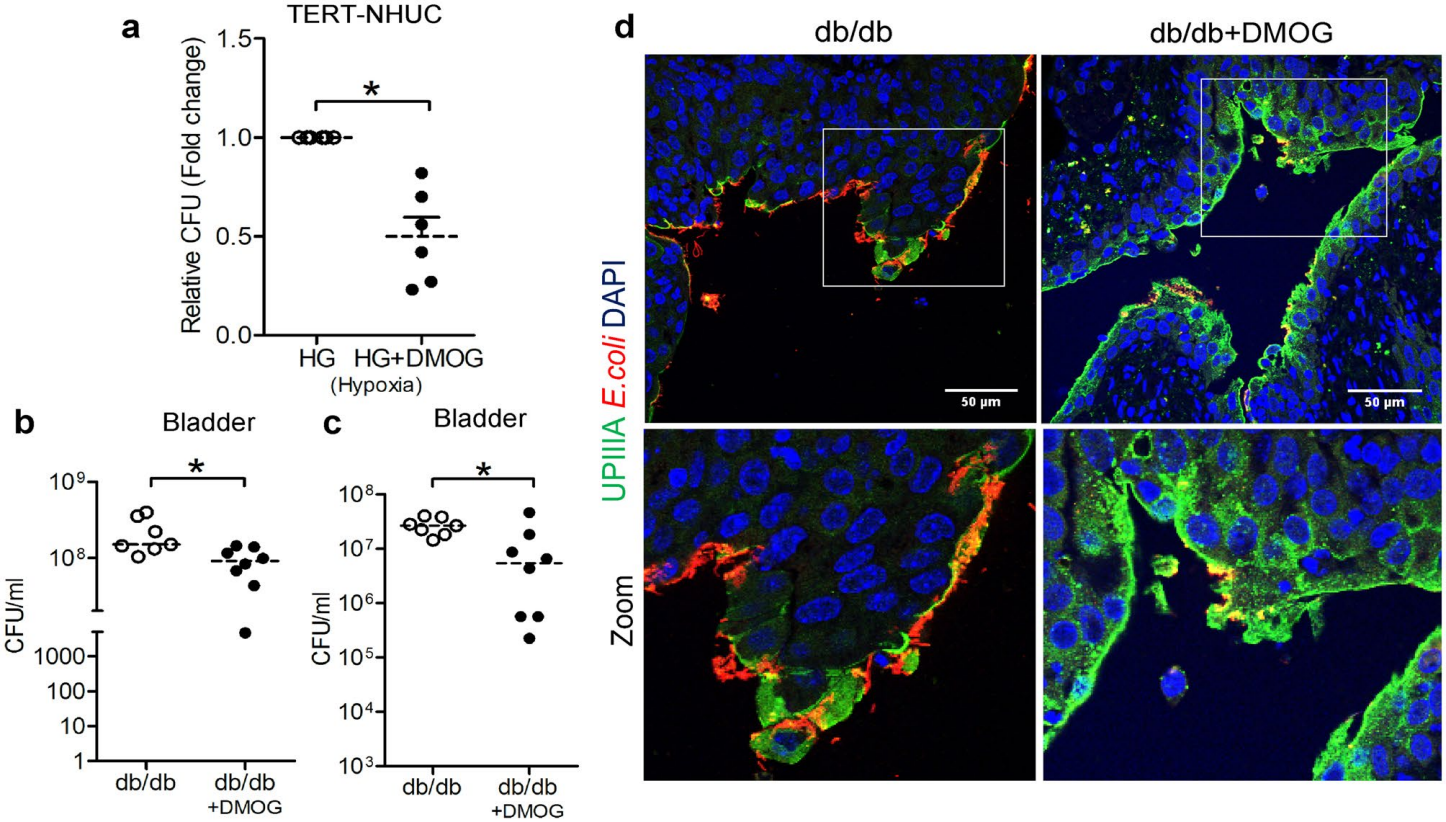
HIF-1 activation decreased bacterial load in diabetic mice and uroepithelial cells. **a** Total number of bacteria in TERT-NHUC cells (*n* = 6, Wilcoxon matched-pairs signed-rank test) 24 h pretreated with vehicle or DMOG and urinary bladder from DMOG-treated diabetes, db/db (*n* = 8) and non-treated (*n* = 7) mice after **b** 24 h and **c** 7 days using CFU, colony-forming unit assay (unpaired *t* test). **d** Representative sections from mouse bladders from 7 days post-infection were stained for UPIIIa (marker of terminally differentiated umbrella cells, green) and *E. coli* (red), with DAPI to show nuclei (blue). *L.p.,* lamina propria; *Lu.,* lumen; *Ep.,* epithelium. In vitro experiments were performed in either duplicate or triplicate. Data are shown as mean ± SEM. Results from mice are presented as median. Significance levels mentioned as *P < 0.05 and ***P < 0.001

The uroepithelial, TERT-NHUC, and 5637 cells exposed to high glucose, hypoxia, and DMOG before *E. coli* infection had reduced bacterial load, compared to cells under the same conditions but without DMOG treatment (Fig. 4a, Fig. S4a). Similarly, diabetic mice, treated with DMOG before transurethral *E. coli* infection, showed lower bacterial load in the bladder compared with vehicle-treated mice at 24 h (Fig. 4b) with a minor but similar trend in the urine (Fig. S4b) and 7 days (Fig. 4c) post-infection. Interestingly, 7 days post-infection, the uroepithelium of all DMOG-treated mice was almost intact while in non-treated mice we observed disruption of the uroepithelial lining with pronounced *E. coli* load (Fig. 4d).

## Discussion

Preventing infections by strengthening the immune response is a tempting alternative to decrease antibiotic consumption. This is particularly important in patients with diabetes because of the frequent serious complications. Since diabetes is known to compromise several antimicrobial peptides like hBD-1, RNase4, RNase7, and LCN2 [16, 28, 29], our results are of special relevance and suggest a mode to reestablish the expression of antimicrobial peptides in type 2 diabetes patients. In normoxia and normal glucose conditions, HIF-1 regulates the expression of LL-37 [5] by binding to its promoter sequence [30]. As LL-37 in the urinary tract is vital to prevent urinary tract infections [31], it is important to secure high levels, especially in patients with diabetes. Moreover, apart from the antimicrobial activity, LL-37 may also regulate islet function and regeneration, thereby promoting glucose homeostasis [32] which may benefit the host during diabetes.

People living at high altitude are known to adapt to hypoxia, and modify the transcriptional response to the HIF pathway [26]. For the first time, we report the association of HIF-1 activation with the antimicrobial peptide LL-37 and HIF-1 target genes *PGK1* and *IL-1B* in type 2 diabetic patients living at high altitude. Previously, a study including 25 non-diabetic individuals of Aymara ancestry from La Paz showed that the HIF pathway was mainly used to activate the genes including *VEGFA* and *IL1B* [33] along with several genes involved in oxygen homeostasis. In other tissues, like the adipose tissue, hypoxia has been shown to induce insulin resistance [34, 35]. Since insulin resistance contributes to increased glucose levels, it cannot be ruled out that the increased HbA1c and urine glucose levels observed among patients from La Paz, in part, were a result of this. In patients with remaining endogenous insulin production, drugs acting on the insulin signaling pathway, like sulfonylurea and GLP-1 agonists, may have an impact on the results. This is particularly relevant since sulfonylurea may also act directly on this pathway by influencing the K_ATP_ channel. However, based on the antidiabetic treatment, we did not observe any difference with respect to *HIF1A* or *CAMP*.

It is well-known that diabetes is more common among men [36] whereas UTI is more frequent in women [37]. We have previously demonstrated the impact of estrogen on uroepithelial cells, showing increased LL-37 [18] and estrogen receptor α, has been shown to directly regulate the HIF-1 pathway [38]. Moreover, studies using rats exposed to chronic hypoxia showed that males were more susceptible to hypoxic pulmonary hypertension and that estrogen was a key factor for protection in females [39].

In patients and mice with diabetes, bacterial clearance is compromised partly because of impaired cytokine expression [27, 40]. Improvement of cytokine expression in diabetes could therefore further enhance the immune response by promoting recruitment of immune cells which in turn could have a beneficial effect on infections. Our results highlighted HIF-1 mediated increased expression of IL-1β and IL-8 even with cells exposed to high glucose. HIF-1 activation in diabetes and in high glucose–treated uroepithelial cells can efficiently restore the expression of proinflammatory cytokines which could benefit the host from invading pathogens. In normoxia, activation with DFO has not been consistent, and either up- or downregulation of IL-1β and IL-8 has been demonstrated [5, 41]. However, since LL-37 is known to upregulate the expression of IL-1β [42], we cannot rule out a direct effect of LL-37 inducing IL-1β which in turn influences IL-8*.*

Furthermore, we highlight the importance of HIF-1 in reducing the bacterial load in high glucose–treated cells and in an in vivo infection UTI model. In concert with our results, non-diabetic people living at high altitude were shown to have decreased mycobacterial growth in whole blood compared to those from low altitude [43] suggesting the involvement of HIF-1 in the antibacterial activity. Our results also support previous findings of activated *Hif1α* upregulation of CRAMP [6] resulting in less *C. albicans* in the gastro-intestinal tract of normoglycemic mice. As CRAMP is highly antibacterial and mice lacking CRAMP are more susceptible to epithelial infections [12, 44], this demonstrates an important role of cathelicidin in innate immunity.

In conclusion, we show an important role of HIF-1 activation in *E. coli* clearance in the presence of high glucose, pointing at a central role of HIF signaling in diabetic patients. We here also demonstrate the impact of high altitude on the immune response in type 2 diabetic patients. The increasing antibiotic resistance is globally a growing concern and fuels a need for alternative treatment strategies. Interest has therefore been focused on antimicrobial peptides. Although there are multiple hurdles, enhancing HIF-1 may, along with antibiotics, in the future complement and strengthen the immunity in selected patient groups where traditional treatment is not possible.

## Supplementary Information

Below is the link to the electronic supplementary material.

Supplementary file1 (TIF 803 KB)

Supplementary file2 (TIF 1122 KB)

Supplementary file3 (TIF 430 KB)

Supplementary file4 (TIF 19010 KB)

Supplementary file5 (DOCX 340 KB)

## Data Availability

Data associated to this manuscript are saved in a data repository.
