# Supplementary figures and images for: HIF-1 mediated activation of antimicrobial peptide LL-37 in type 2 diabetic patients
Source: J Mol Med (Berl). 2021 Oct 15;100(1):101–13. doi: 10.1007/s00109-021-02134-7 (PMC8724101; doi:10.1007/s00109-021-02134-7)

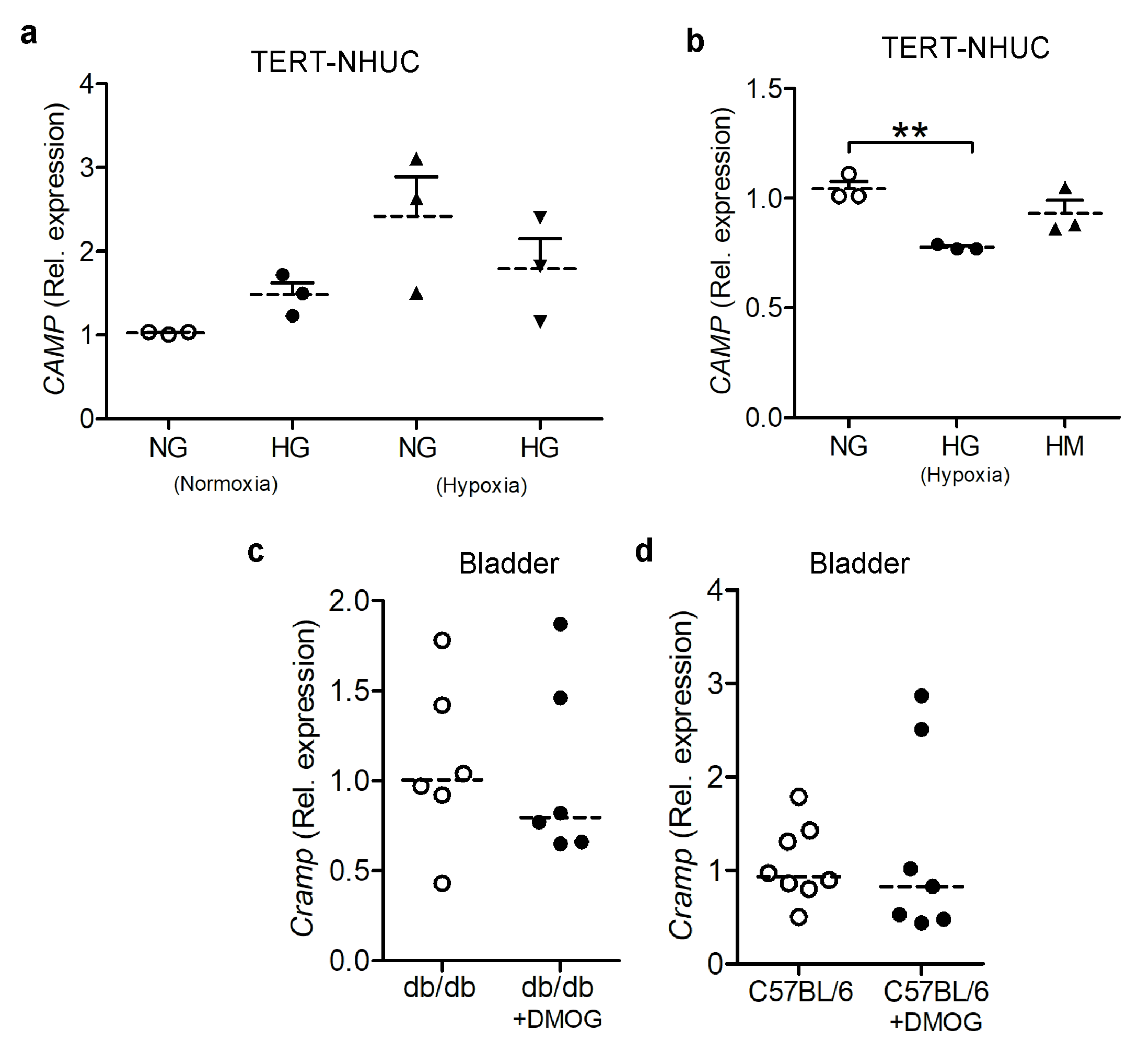

Supplement: Supplementary file 1 — Supplementary file1 (TIF 803 KB) [file 109_2021_2134_MOESM1_ESM.tif]

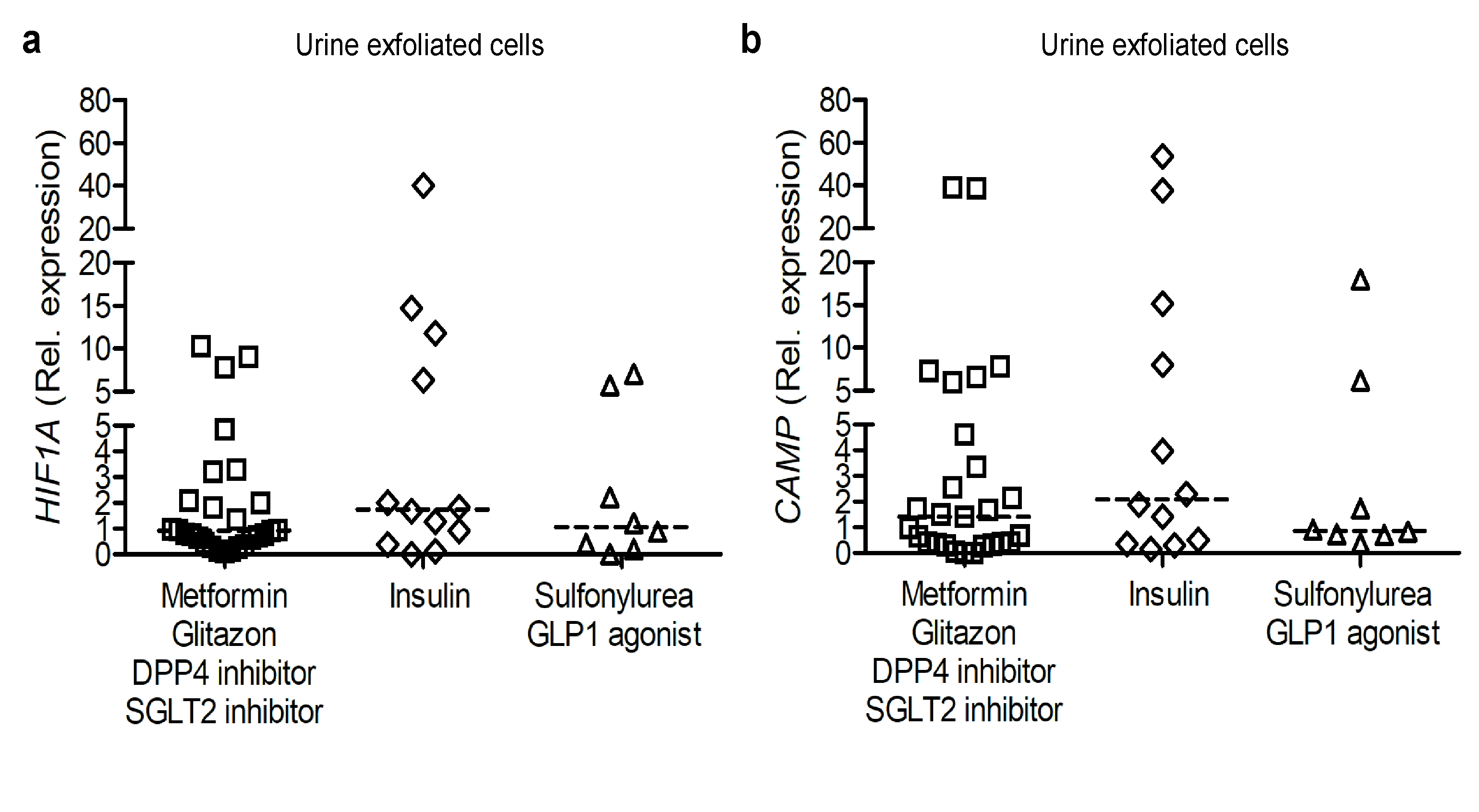

Supplement: Supplementary file 2 — Supplementary file2 (TIF 1122 KB) [file 109_2021_2134_MOESM2_ESM.tif]

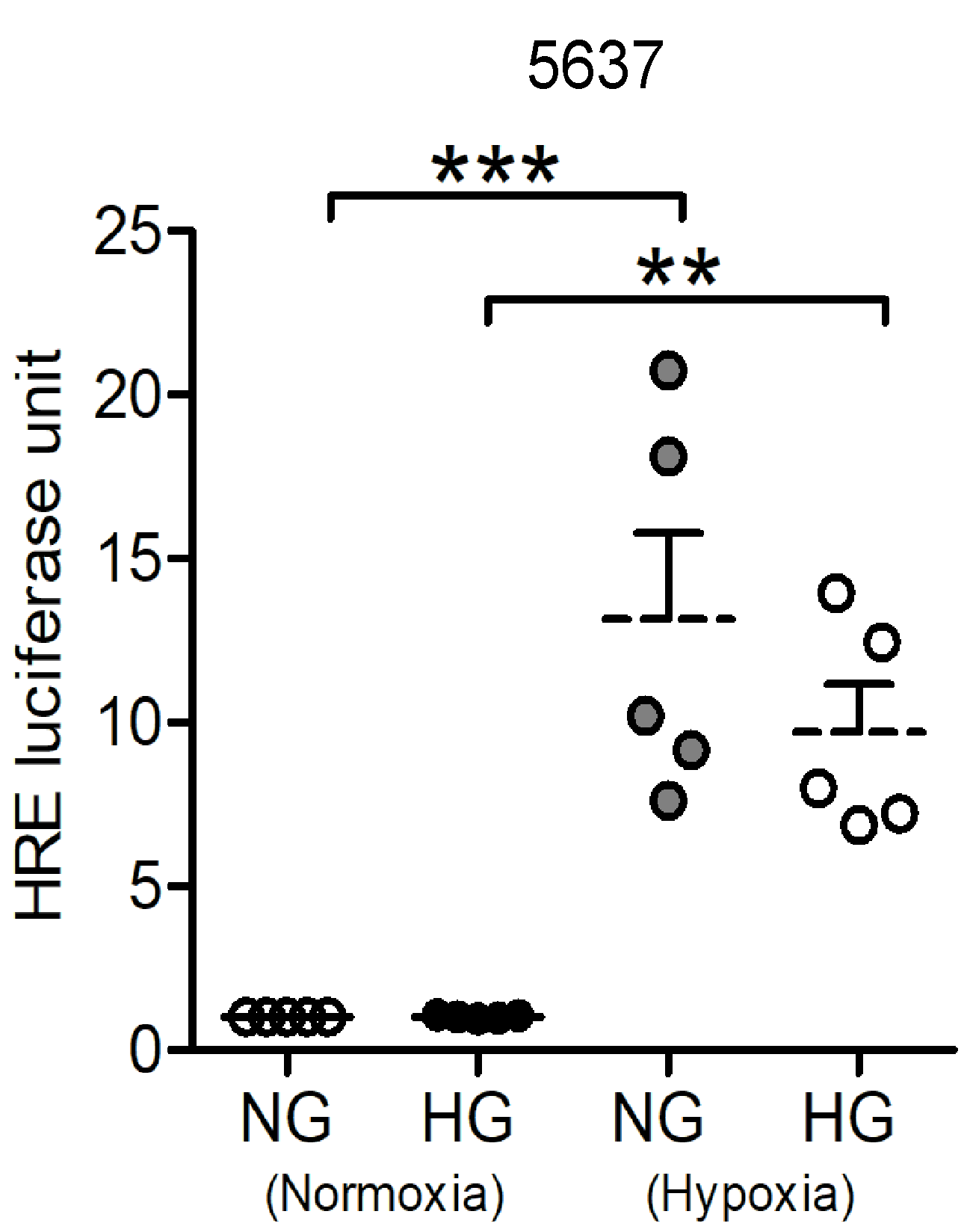

Supplement: Supplementary file 3 — Supplementary file3 (TIF 430 KB) [file 109_2021_2134_MOESM3_ESM.tif]

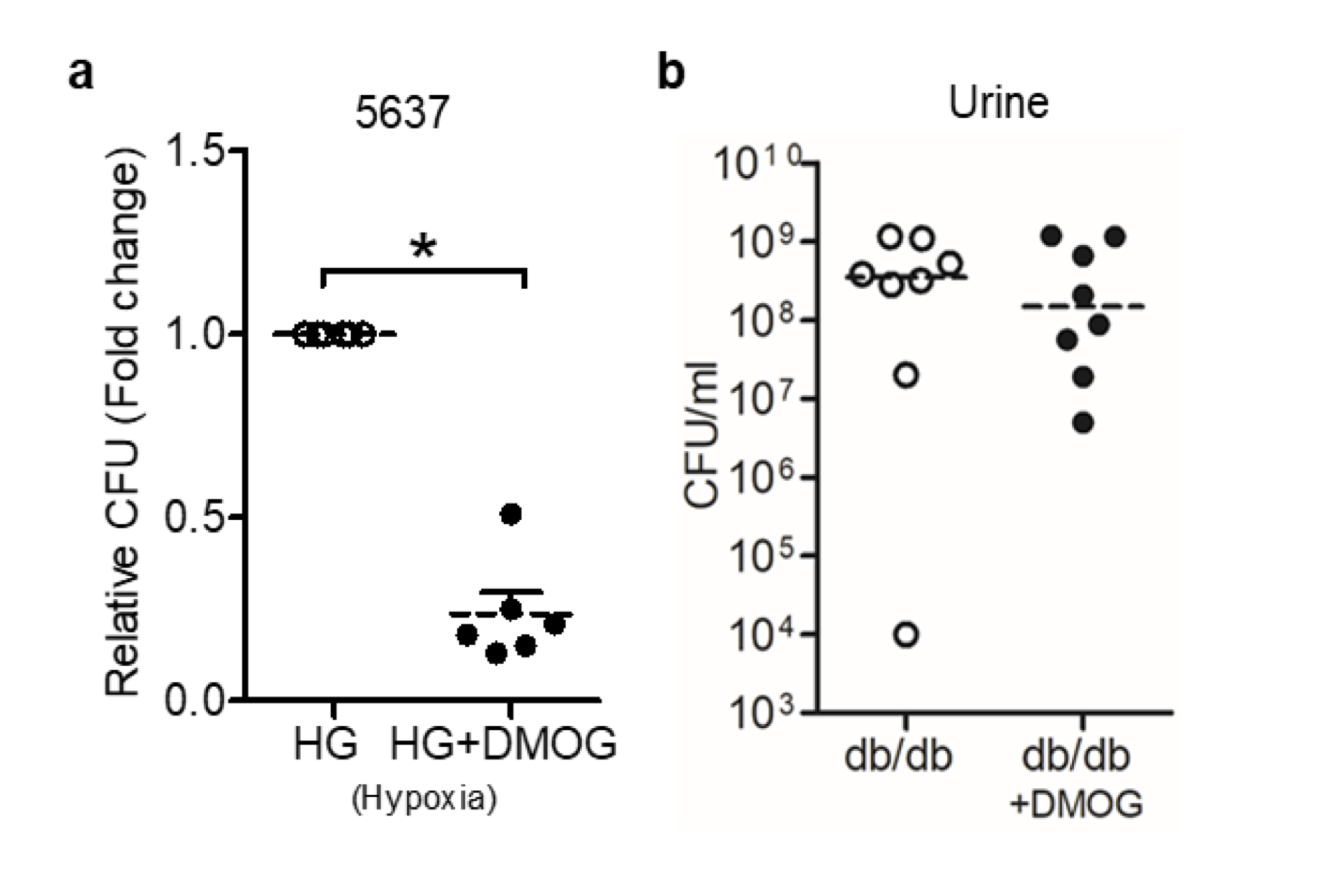

Supplement: Supplementary file 4 — Supplementary file4 (TIF 19010 KB) [file 109_2021_2134_MOESM4_ESM.tif]
