# Supplementary material for: HIF-1 mediated activation of antimicrobial peptide LL-37 in type 2 diabetic patients
Source: J Mol Med (Berl). 2021 Oct 15;100(1):101–13. doi: 10.1007/s00109-021-02134-7 (PMC8724101; doi:10.1007/s00109-021-02134-7)
Supplement: Supplementary file 5 — Supplementary file5 (DOCX 340 KB) [file 109_2021_2134_MOESM5_ESM.docx]

***Supplementary information***

**(JMME-D-21-00111)**

**HIF-1 mediated activation of antimicrobial peptide LL-37 in type 2 diabetic patients**

Soumitra Mohanty^1^, Witchuda Kamolvit^1^, Silvia Zambrana^2,3^, Eduardo Gonzales^3^, Jonas Tovi^4^, Kerstin Brismar^2^, Claes-Göran Östenson^2^, and Annelie Brauner^1^*

^1^ Department of Microbiology, Tumor and Cell Biology, Division of Clinical Microbiology,

Karolinska Institutet and Karolinska University Hospital, Stockholm, Sweden

^2^ Department of Molecular Medicine and Surgery, Karolinska Institutet, Stockholm, Sweden

^3^ Area de Farmacologia, Instituto de Investigaciones Farmaco Bioquimicas, Facultad de Ciencias Farmacéuticas y Bioquimicas, Universidad Mayor de San Andres, La Paz, Bolivia

^4^ Capio Health Care Center, Solna, Sweden

* Corresponding author:

Annelie Brauner, Department of Microbiology, Tumor and Cell Biology, Division of Clinical Microbiology, Karolinska Institutet and Karolinska University Hospital, 17176 Stockholm, Sweden.

Phone +46 8 51770000, Fax: +46 8 308099

E-mail: [Annelie.Brauner@ki.se](mailto:Annelie.Brauner@ki.se)

**Supplementary fig.1**

**
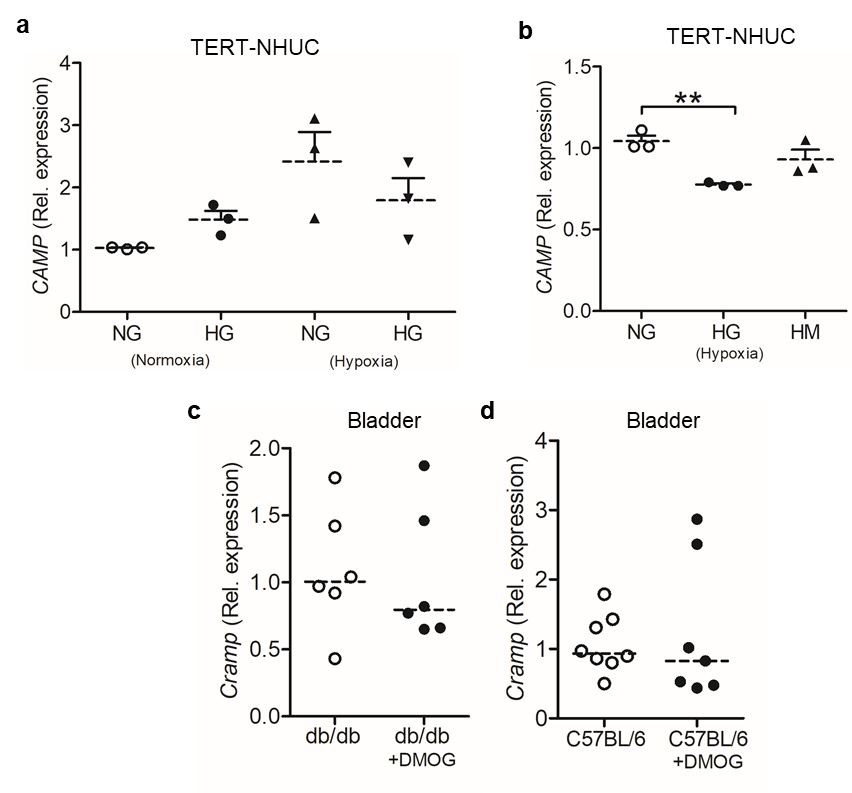
**

**Supplementary fig. 1 Expression of antimicrobial peptide, LL-37.** TERT-NHUC cells, demonstrating expression of *CAMP* mRNA 24 h post treatment of **(a)** glucose (n=3) and **(b)** mannitol (n=3, Bonferroni’s multiple comparison test) in both normoxia and hypoxia. *Cramp* mRNA in DMOG or vehicle (DMSO) treated uninfected **(c)** type 2 diabetic, db/db (n=6 each) and **(d)** nondiabetic C57BL/6 mice urinary bladder (n=8 each) after 7 days. *In vitro* analysis was performed in duplicate. Average values are shown for each set. Normal glucose; 6mM (NG), high glucose; 30mM (HG), High mannitol; 30mM (HM), normoxia; 21% O_2_ (N) and hypoxia; 1% O_2_ (H). Data are shown as mean + SEM. Results from mice are presented as median. Significance levels mentioned as ***P* < 0.01.

**Supplementary fig.2**

**
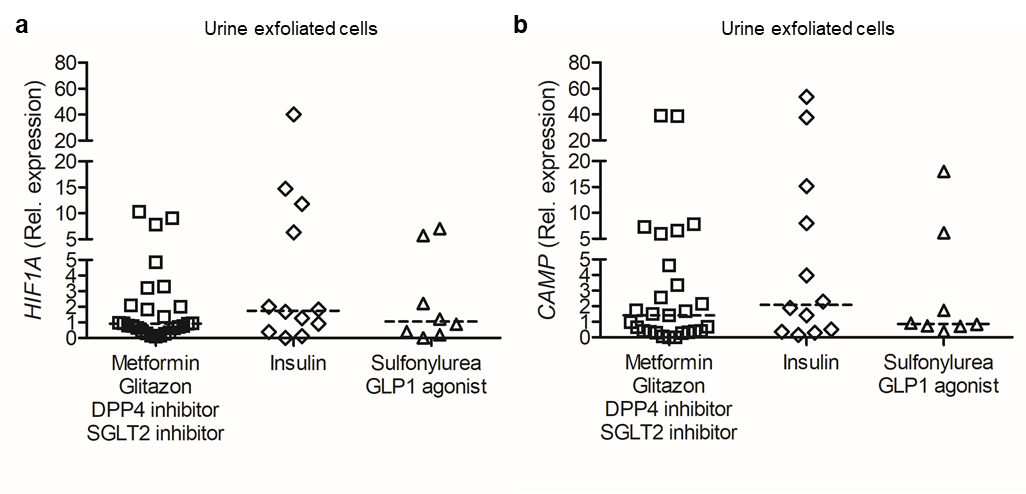
**

**Supplementary fig. 2 Impact on *HIF1A* and *CAMP* by insulin and drugs affecting the insulin signaling pathway compared to other antidiabetic drugs.** Human urine exfoliated cells from type 2 diabetic patients (T2D) living at low or high altitude were analyzed for **(a)** *HIF1A* **(b)** *CAMP* mRNA and grouped based on the antidiabetic treatment. Other antidiabetic drugs: metformin/glitazon/DPP4 inhibitor/SGLT2 inhibitor (n=27); insulin (n=12); Drugs affecting the insulin signaling pathway: sulfonylurea/GLP-1 agonists (n=8).

**Supplementary fig. 3**


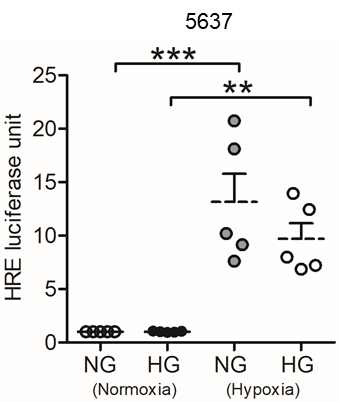


**Supplementary fig. 3 High glucose impairs hypoxia induced luciferase activity.** Uroepithelial cells, 5637, demonstrating expression of HRE luciferase assay 39 h post treatment of glucose in both normoxia and hypoxia (n=3, Bonferroni’s multiple comparison test). This analysis was performed in either duplicate or triplicate. Average values are shown for each set. Normal glucose; 5.5 mM (NG), High glucose; 30mM (HG), normoxia; 21% O_2_ and hypoxia; 1% O_2_. Data are shown as mean + SEM. Significance levels mentioned as ***P* < 0.01 and ****P* < 0.001.

**Supplementary fig.4**


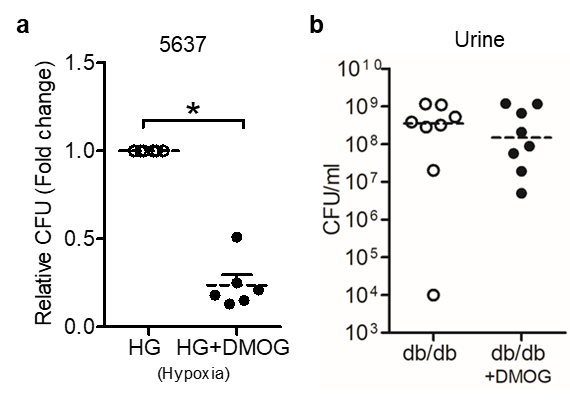


**Supplementary fig. 4 HIF-1 activation decreased bacterial load in uroepithelial cells and diabetic mice.** **(a)** Total number of bacteria in 5637 cells, 24 h after pretreatment with vehicle or DMOG (n=6, Wilcoxon matched pairs signed rank test). **(b)** Urine CFU, colony forming unit assay from DMOG treated diabetes, db/db and non-treated (n=8 each) mice after 24 h with or without DMOG treatment. *In vitro* experiments were performed in duplicate or triplicate. Average values are shown for each set. High glucose (HG). Data are shown as mean + SEM. Results from mice are presented as median. Significance levels mentioned as **P* < 0.05.
